# Father and son with a pathogenic variant c.614dup p.(Gln206Thrfs*20) in the *NR5A1* gene: a case report

**DOI:** 10.3389/fped.2026.1946666

**Published:** 2026-09-18

**Authors:** Júlia Martinková, Michaela Mihulová, Miroslava Balaščaková, Jan Trachta, Anna Křepelová

**Affiliations:** 1 Department of Biology and Medical Genetics, Second Faculty of Medicine, Charles University in Prague, Motol and Homolka University Hospital, Prague, Czechia; 2 Department of Paediatric Surgery, Second Faculty of Medicine, Charles University in Prague, Motol and Homolka University Hospital, Prague, Czechia

**Keywords:** disorders of sex development, IVF, male phenotype, *NR5A1* duplication, testicular dysgenesis

## Abstract

**Background:**

Steroidogenic factor 1 (SF-1), encoded by the *NR5A1* gene, is a critical transcriptional regulator of adrenal and gonadal development. Pathogenic *NR5A1* variants lead to a broad phenotypic spectrum characterized by insufficient virilization, including gonadal and testicular dysgenesis, ambiguous genitalia, hypospadias, micropenis, cryptorchidism, anorchia, and male infertility. This case report characterizes the clinical and genetic findings in a boy and his father with differences of sex development (DSD) caused by a heterozygous *NR5A1* frameshift variant, thereby expanding our understanding of phenotypic mechanisms, paternal inheritance, and reproductive potential in this disorder.

**Case presentation:**

We report a unique case of a Czech family in which a father and his son exhibited a 46,XY karyotype, genital malformations, and palpable testes. Exome sequencing identified the heterozygous frameshift variant, c.614dup p.(Gln206Thrfs*20), in the *NR5A1* gene in both individuals. This rare *NR5A1* variant had previously been reported only in 46,XY female patients with DSD.

**Conclusion:**

Here, we present the first report of paternal transmission of the pathogenic NM_004959.5(NR5A1):c.614dup p.(Gln206Thrfs*20) variant to an affected son. Despite the associated genital malformation and the genetic variant, the father achieved successful reproduction via *in vitro* fertilization.

## Introduction

Disorders or differences of sex development (DSD) are conditions where the development of chromosomal, gonadal, or anatomical sex is atypical (1–3). DSD are categorized into three groups: 46,XY DSD, 46,XX DSD, and chromosomal DSD. The 46,XY DSD group is further divided into three categories: (a) disorders of gonadal (testicular) development, including complete or partial gonadal dysgenesis, ovotesticular DSD (OT-DSD), and gonadal regression; (b) disorders in androgen synthesis or action; and (c) others (3). 46,XY gonadal dysgenesis was initially named “Swyer syndrome” (MIM:400044; 46,XY Sex reversal 1), after Gerald Swyer, who first described this condition (4). It is among the most common groups, frequently caused by disruption of the *SRY* gene (MIM:*480000), with an estimated incidence of 1:80,000 in newborns (5). Other groups include 46,XY Sex reversal 3 (MIM:612965) and 46,XX Sex reversal 4 (MIM:617480), caused by pathogenic variants in the *NR5A1* gene (MIM:*184757), which exhibit autosomal dominant inheritance. Variants in *NR5A1* have been identified in approximately 10%–20% of individuals with 46,XY DSD, corresponding to an incidence of roughly 1:100,000–1:250,000 newborns (6, 7). Additional genes, including *MAP3K1, SOX9, WT1, WNT4, GATA4, DHH, FOG2, FGF9, ZNRF3, PBX1, ATRX, DMRT1, CBX2, NR0B1, DHX37,* and *SOX8*, may also contribute to gonadal dysgenesis in individuals with 46,XY DSD (8, 9).

Disruptions in *NR5A1* cause various forms of insufficient virilization in individuals with 46,XY DSD, such as gonadal and testicular dysgenesis, ambiguous genitalia, hypospadias, micropenis, cryptorchidism, anorchia, and male infertility (MIM:613957) (10, 11). 46,XX individuals with normal female genital development and an *NR5A1* variant have been diagnosed with premature ovarian insufficiency (POI) (MIM:612964) (12). Phenotypic variability, whether with different or identical *NR5A1* variants, has been observed among individuals within familial clusters (13). Consequently, establishing a direct phenotype–genotype correlation remains difficult, resulting in a broad spectrum of phenotypes (14).

The *NR5A1* gene, situated on chromosome 9q33, comprises one noncoding exon 1 and six coding exons, namely 2**–**7 (15). It encodes a nuclear receptor subfamily 5 group A member 1 (NR5A1), also known as steroidogenic factor 1 (SF-1) (16, 17). *NR5A1* features several functional domains: a DNA-binding domain (DBD), which includes two zinc fingers and an FTZ-F1 box; a hinge region; a ligand-binding domain (LBD); and two activation domains, AF-1 and AF-2 (18). Research indicates that *NR5A1* also localizes to centrosomes, where it plays a role in maintaining genomic stability by regulating centrosome homeostasis (19).

*NR5A1* influences male development by regulating genes involved in steroid production, adrenal and gonadal growth, reproduction, and energy metabolism (20). In Sertoli cells, it controls the transcription of *SOX9* (MIM:*608160), with synergistic activity of the *SRY* gene. This process leads to the expression of Anti-Müllerian hormone (AMH) and the regression of Müllerian structures (21, 22). In Leydig cells, NR5A1 regulates the expression of the luteinizing hormone choriogonadotropin receptor (*LHCGR*) and steroidogenic enzymes such as STAR*,* CYP11A1, and CYP17A1, resulting in masculinization of the external genitalia (21, 23). NR5A1 also enhances the expression of insulin-like polypeptide 3 (INSL3, MIM:*146738), which is crucial for testicular descent (24). In females, NR5A1 interacts with and modulates the WNT4/*β*-catenin pathway, vital for female sex differentiation and ovarian development, supporting ovarian follicle reserve formation, oogenesis, and overall ovarian function (25, 26).

This report describes a unique 46,XY patient presenting with a micropenis, hypospadias, and palpable testes, who harbors a confirmed *NR5A1* pathogenic variant inherited from his affected father. This finding is supported by a review of the literature concerning 46,XY individuals with DSD carrying this specific *NR5A1* variant.

## Case presentation

The proband is a 3-year-old male patient (III:2) who exhibited dysmorphic genitalia, a bifid scrotum, palpable testes, and developed micropenis with proximal-perineal hypospadias. The child was from the third pregnancy, delivered on time (39th week), with a birth weight of 3380 g and a length of 51 cm. The patient was conceived via *in vitro* fertilization (IVF) due to oligospermia in the patient's father (II:1). The genital appearance of the proband showed greater resemblance to female sex (Figure 1B), with a description of ambiguous genitalia: labioscrotal ridges, bilateral palpable testes, hypoplastic penis, and penoscrotal transposition. The proband was found to have a 46,XY karyotype postnatally. The hormonal profile of the 2-month-old patient is outlined below: testosterone (T) 1.29 nmol/L (ref. range 0.24−0.36 nmol/L), 17-hydroxyprogesterone (17-OHP) 5 nmol/L (1.27-8.80 nmol/L), adrenocorticotropic hormone (ACTH) 69 ng/L (5-46 ng/L), dehydroepiandrosterone (DHEA-S) 0.77 μmol/L (0.7-15.6 μmol/L), androstenedione 1.3 nmol/L, aldosterone 420 ng/L (50-900 ng/L), cortisol (F) 369 nmol/L (28.0-550.0 nmol/L). At 2 months, his weight was 6,100 g (91st percentile, +1.3 SD), and length was 59 cm (71st percentile, +0.6 SD). Testosterone levels were repeatedly assessed during the first weeks of life and were within the normal range. At 1 year and 2 months, his weight was 12 kg, height 80 cm, and head circumference 50 cm. At 1 year and 4 months, a genital surgery was performed. Bracka's two-stage plastic surgical approach (27) was considered the best option. The appearance of the genitalia following these procedures is illustrated in Figure 1. The surgical procedures were performed before the genetic examination of the patient. After the initial surgeries, the 3-year-old proband and his parents were available for clinical and molecular genetic assessment. At 3 years and 9 months, his height was 107.6 cm (0.8 SD)—taller than average, with growth within the normal range. His estimated target height, based on his parents' heights of 170.1 cm (mother) and 172.0 cm (father), is approximately 177.6 ± 8.5 cm. His weight was 18 kg (0.6 SD), and BMI was 15.5 (−0.1 SD), reflecting normal nutritional status. Sexual maturation was classified as P1, with testes measuring 1 mL in volume bilaterally. The hypospadias and the bifid scrotum were corrected surgically. Ultrasound examination did not detect a uterus. Sonography of the central nervous system and abdomen was unremarkable. Psychomotor development was normal at 3 years and 9 months, consistent with age, with no distinct toy preferences. The patient has consistently achieved developmental milestones within age-appropriate norms.

**Figure 1:**
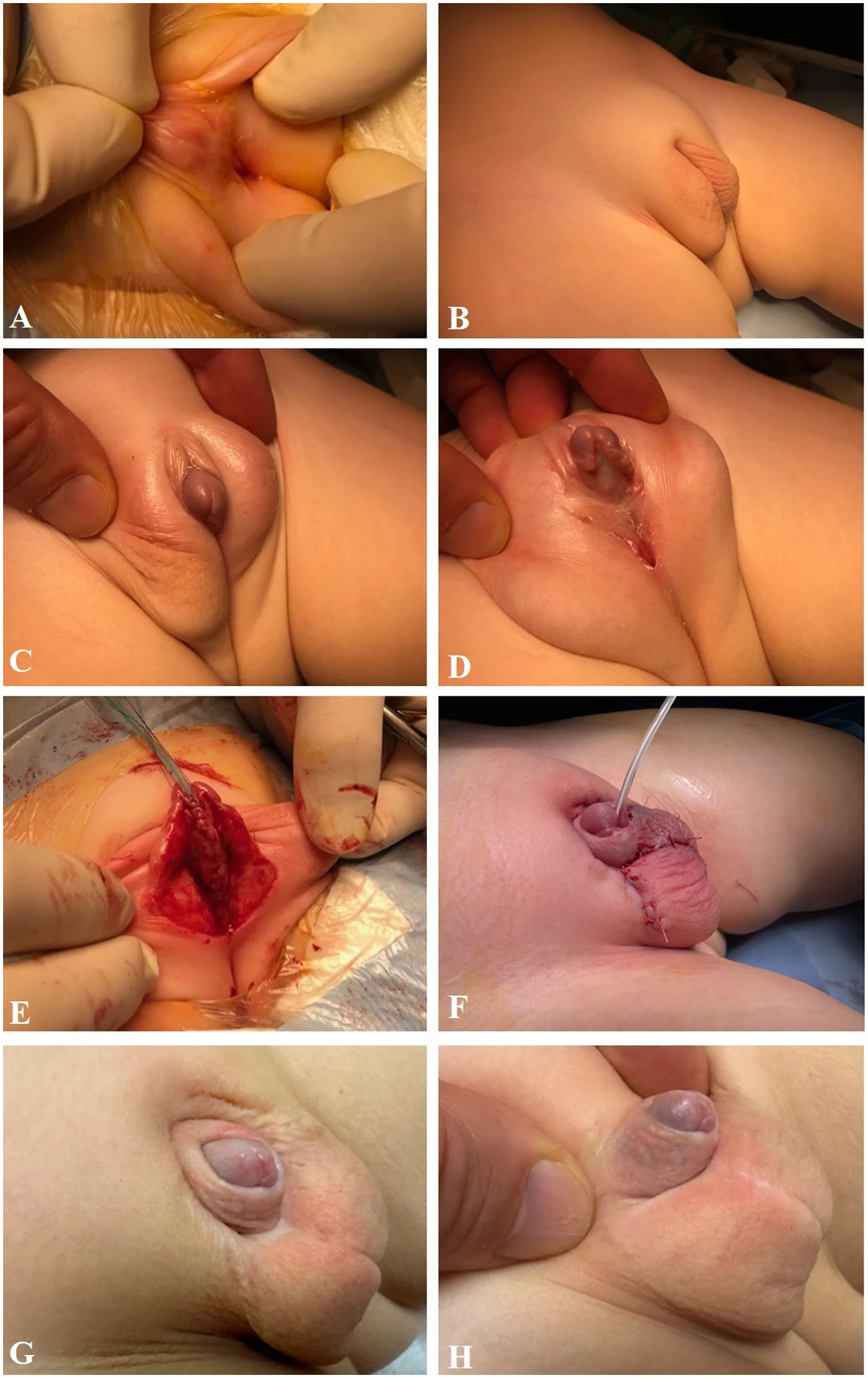
Patient with perineal hypospadias. **(A, B)** Ventral angulation of the smaller penis by 90 degrees, and a glans, pulled by a fibrous cord to the perineal meatus of the proximal urethra. The first phase of a two-stage reconstructive operation, according to Bracka, was performed, in which the skin graft taken from the shortened foreskin was sutured to the ventral side of the straightened penis. **(C, D, E)** Six months after healing of the first phase, the external appearance of the genitals remains unchanged, but after stretching the scrotal folds, a straightened, smaller penis with a well-attached graft is visible, ready for closure and urethral reconstruction in the second phase. **(F)** After healing of the urethra and the entire penis, the final, third stage of the operation remains, which is correction of peno-scrotal transposition - pulling the scrotum with the testicles under the penis into a more physiological position. **(G, H)** After another six months of healing, the genitals are reconstructed, and the boy urinates without straining, with a strong stream through a meatus at the end of his penis. He will also be able to have straight erections in the future with no ventral angulation of the penis.

The patient's parents are non-consanguineous. The patient's father (II:1) exhibited a similar phenotype, but he underwent several genital surgeries during childhood and adolescence without prior genetic counseling or molecular genetic analysis. Therefore, photodocumentation or a detailed description of the phenotype is not available. In the medical records, his condition is only briefly described as hypospadias. For the purpose of fertility preservation, the sperm sample from the patient's father was obtained and preserved when he was approximately 15 years old. The patient has a healthy 5-year-old sister (III:1), who was also conceived through IVF. The patient's oldest healthy half-sister, aged 20, is biologically related only through the maternal side. The patient's father has one adult sister without evidence of gonadal dysgenesis; she is cured of endometriosis, and she has one healthy son. The patient's paternal grandparents and their siblings, along with their descendants, are reported to be healthy. Once the patient's younger sister reaches reproductive age, clinical-genetic counseling is recommended. Examination of the patient's paternal aunt, who is still of reproductive age, is also recommended.

## Materials and methods

### Editorial policies and ethical considerations

The consent form received approval from the Ethics Committee of Motol University Hospital, Prague, Czech Republic. All procedures were carried out in accordance with the principles outlined in the World Medical Association Declaration of Helsinki. The patient underwent molecular genetic analysis after obtaining written informed consent from the patient's parent.

### Molecular genetic methods and data analysis

The patient's genomic DNA was extracted from leukocytes in peripheral blood following a standard procedure using the MagCore® Super Automated Nucleic Acid Extractor (RBC Bioscience Corp., New Taipei City, Taiwan). Whole exome sequencing was carried out with Illumina's Twist Comprehensive Exome and Twist Mitochondrial DNA panel on a NovaSeq 6000 (Illumina, USA) at the Institute of Applied Biotechnologies a.s. (IAB; Olomouc, Czech Republic) in trio mode (with parents). The reference genome GRCh37/hg19 served as a basis for sequence comparison. Single-nucleotide variants (SNVs) and copy-number variations (CNVs) were annotated, filtered, and interpreted using the Franklin platform by Genoox software (franklin.genoox.com, Israel). A virtual panel comprising 130 genes linked to DSD was used to identify pathogenic and likely pathogenic variants (Supplementary Material 1). Filtering involved assessing variant frequency in the general population (gnomAD, dbSNP databases), presence in clinical databases (ClinVar, Human Gene Mutation Database—HGMD), familial segregation with the phenotype, and *in silico* predictions via bioinformatics tools integrated in VarSome Premium software (varsome.com, Saphetor, Switzerland). The pathogenicity of variants was evaluated following the guidelines of the American College of Medical Genetics and Genomics (ACMG) and the Association for Molecular Pathology (AMP) (28). Results were confirmed through polymerase chain reaction (PCR) and targeted Sanger sequencing on an ABI 3130xl Genetic Analyzer (Applied Biosystems, Foster City, CA, USA) using a BigDye Terminator v3.1 Cycle Sequencing Kit (Applied Biosystems), following the manufacturer's protocol in both directions. Electropherograms were analyzed using Chromas Lite MFC Application, v.2.6.6 (Technelysium, Australia), and Alamut Visual Plus software, v.1.6.1 (SOPHiA GENETICS, Switzerland).

## Results

We identified a heterozygous pathogenic variant, NM_004959.5(NR5A1):c.614dup p.(Gln206Thrfs*20), in exon 4 of the *NR5A1* gene (rs1234904066, Variation ID: 2443198) in the proband (III:2) and his father (II:1) (Figure 2, Figure 3). The variant was not detected in the unaffected mother (II:2). This single-base-pair frameshift duplication results in a premature termination codon at position 225 in the hinge region. This sequence change is predicted to produce an abnormal transcript, which may be degraded by nonsense-mediated decay or lead to the production of a truncated NR5A1 protein with potentially abnormal function (13, 29).

**Figure 2:**
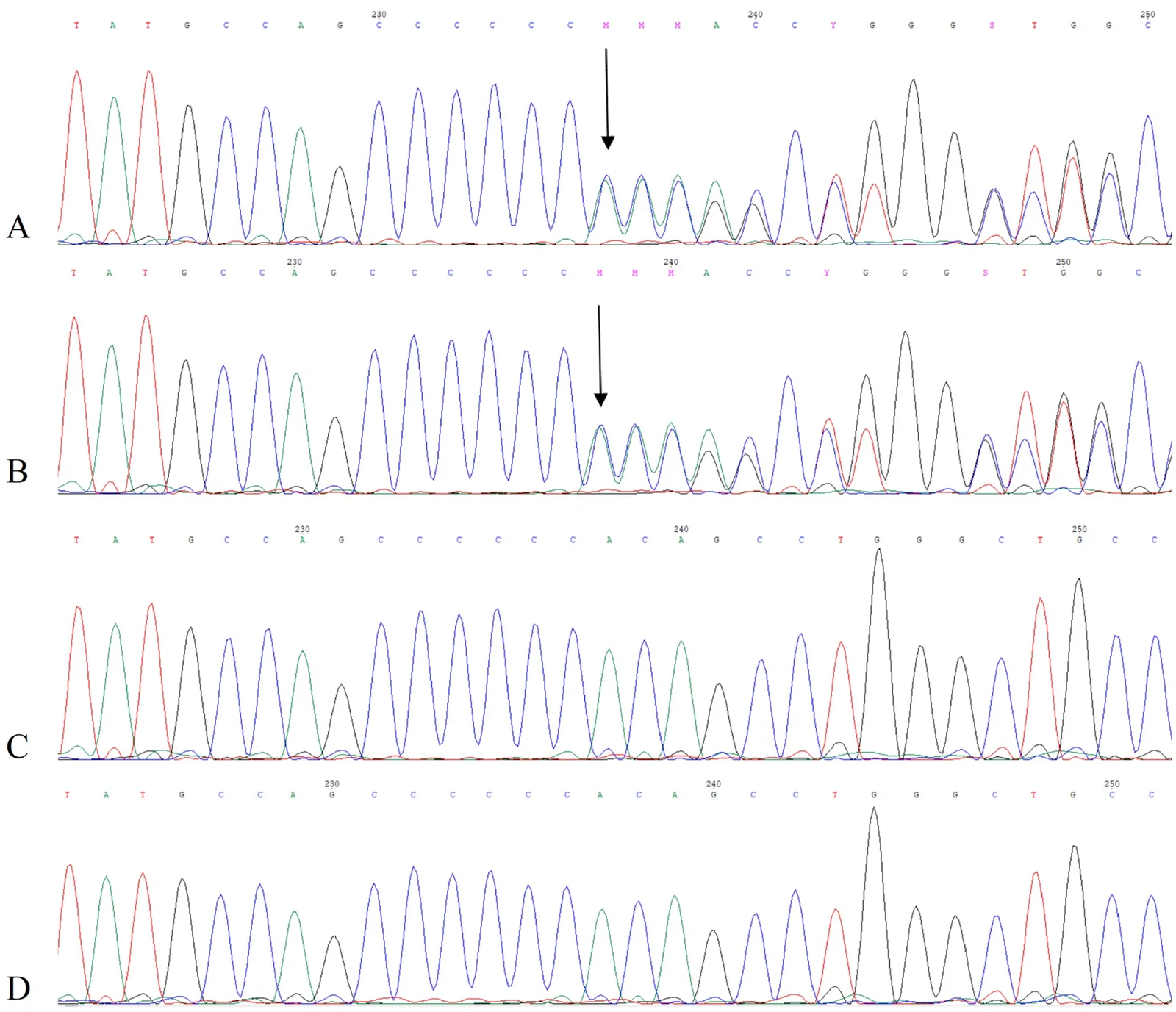
Genetic analysis of the *NR5A1* gene. Partial electropherogram of exon 4 of the *NR5A1* gene, showing the NM_004959.5(NR5A1):c.614dup p.(Gln206Thrfs*20) variant indicated by a black arrow in the affected family members: **(A)** proband; **(B)** proband's father; **(C)** proband's unaffected mother; **(D)** control sequence. Sequencing data were visualized using Chromas Lite MFC Application v.2.6.6 (Technelysium). All electropherograms represent the forward strand.

**Figure 3:**
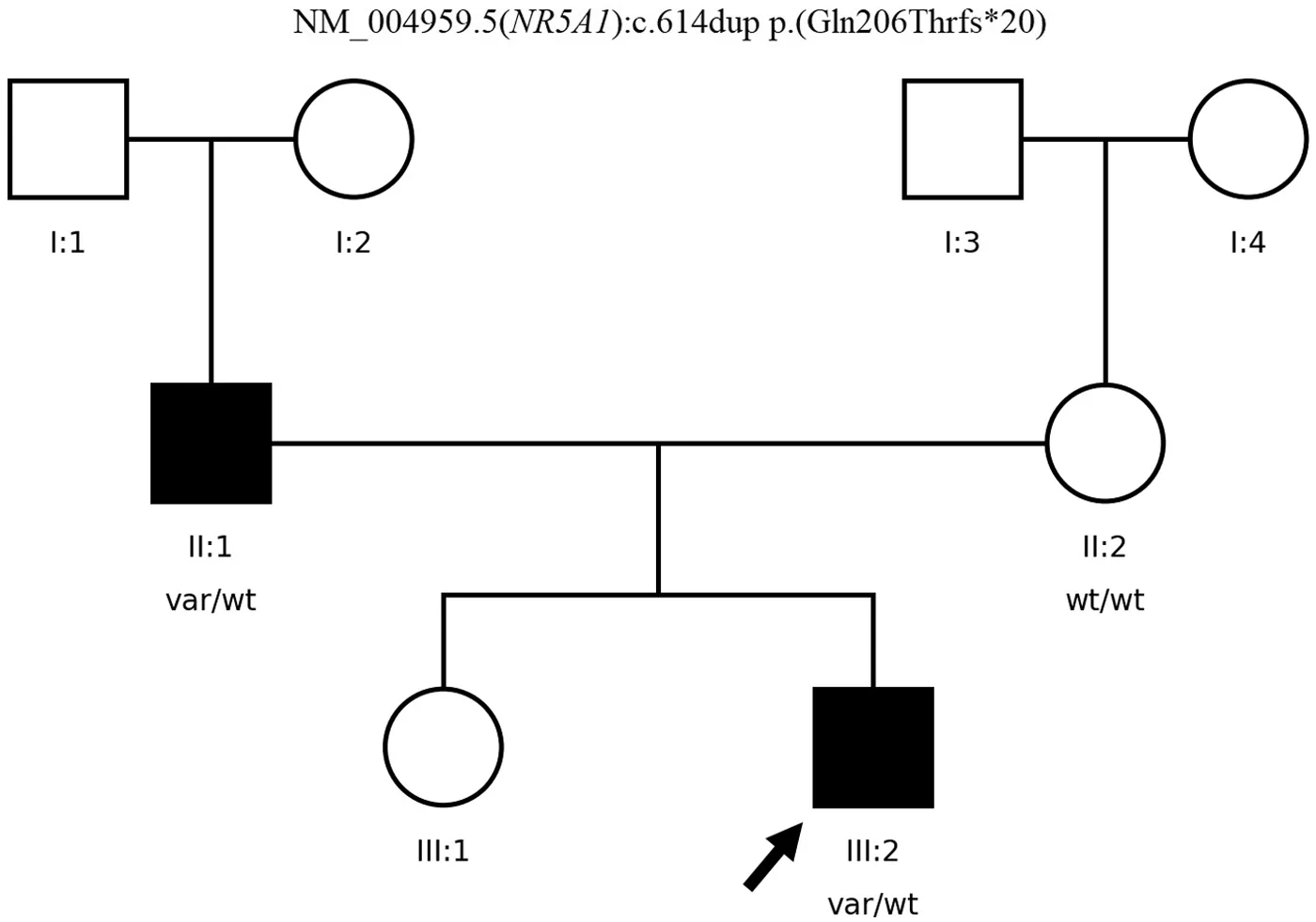
Family pedigree. A three-generation pedigree of the family demonstrating the segregation of the NM_004959.5(NR5A1):c.614dup p.(Gln206Thrfs*20) variant. The proband is indicated by an arrow (III:2). Fully darkened symbols represent affected family members with a 46,XY karyotype (II:1, III:2). The mother of the proband, with a 46,XX karyotype, tested negative for the *NR5A1* variant (II:2). The remaining family members (I:1, I:2, I:3, I:4, III:1) were not molecularly tested.

This *NR5A1* variant has previously been classified as pathogenic in several publications (23, 29, 30) and has also been reported by Martínez de LaPiscina et al. (31) and Camats et al. (13). It is currently listed once as likely pathogenic in the ClinVar database. The variant's frequency is not documented in population databases (gnomAD Exomes, v.4.1; gnomAD Genomes, v.4.0). Although *in silico* prediction tools within the VarSome platform were unavailable, the variant was classified as likely pathogenic by both the Franklin (Genoox) and VarSome Premium platforms. Functional studies have not been performed. Consequently, we classified this variant as pathogenic (criteria: PVS1, PM2, PP1, PP4, PP5) according to the ACMG guidelines (28).

## Discussion

In this report, we describe a pathogenic variant, c.614dup p.(Gln206Thrfs*20), in the *NR5A1* gene in the patient and his father. This *NR5A1* variant has previously been reported as c.614_615insC p.Gln206ThrfsX20 across several publications. However, all five previously reported patients were identified as 46,XY females with DSD (13, 23, 29–31), with two out of the five cases having undergone gonadectomy (29, 31). These patients exhibited a wide phenotypic spectrum (23), including primary infertility (29), primary amenorrhea (29), a hypoplastic uterus (29, 31), or an absent uterus (23), without ovaries (23, 31). Associated findings included hypoplastic/dysplastic testes (23, 30, 31), palpable gonads (13, 29), and the absence of Müllerian structures (13, 23, 31). Normal female external genitalia were explicitly reported in previous cohorts (13, 29–31). Detailed clinical characteristics, including a comparison of hormonal levels and phenotypic features with previously published patients, are presented in Supplementary Material 2. Our patient with a 46,XY karyotype exhibited genital malformations, including dysmorphic genitalia, a bifid scrotum, palpable testes, a micropenis, and hypospadias. His biological father exhibited a similar phenotype prior to genital surgery. Additionally, the father's spermiogram showed evidence of spermatogenesis and sperm motility, though at a low level.

Both sporadic, *de novo,* and inherited cases of *NR5A1* variants have been well documented (13). Most reported *NR5A1* variants are *de novo* or maternally inherited (23). However, a few documented cases of paternal inheritance exist, attributed to incomplete penetrance (16, 23, 32) or mosaicism (33), where the fathers remained unaffected. Nonetheless, Ciaccio et al. (34) reported a different *NR5A1* variant, transmitted by an affected father with a milder phenotype, displaying hypospadias, to his two affected 46,XY sons and two 46,XX daughters with premature ovarian insufficiency. Similarly, Globa et al. (35) reported two male siblings (Patients 12 and 13) where paternal transmission of an *NR5A1* splice-site variant was expected. These boys were conceived via IVF, and they exhibited bilateral cryptorchidism, perineal hypospadias, and micropenis. Their clinical history indicated that both their father and grandfather had exhibited similar phenotypes at birth and had undergone genital surgery. To date, only *de novo* cases of the NM_004959.5(NR5A1):c.614dup p.(Gln206Thrfs*20) variant have been reported (23, 29); a publication by Martínez de LaPiscina et al. (31) investigated only the patient's mother, who was a non-carrier. Here, we present the first report of paternal inheritance of this *NR5A1* variant. The variant is located in the hinge region and is predicted to produce a truncated NR5A1 protein lacking the LBD, leading to NR5A1 inactivation or degradation by nonsense-mediated decay (13, 29). By 2020, more than 180 pathogenic *NR5A1* variants had been documented in the literature (16). The variant spectrum is predominantly composed of missense mutations (58%), followed by a combined 24% of in-frame and frameshift alterations, 12.3% nonsense mutations, 3.3% intronic variants, and less than 1% extension variants. The majority of these variants are located in the LBD (42.3%) and the DBD (35%) domains in both 46,XY and 46,XX individuals (16). Variants in the DBD consistently impair transactivation activity more than those in the LBD or hinge region, although with differing consequences (13). However, the mutational landscape of *NR5A1* is continuously evolving; according to the HGMD Professional database (version 2026.4), the total number of recognized pathogenic (disease-causing) variants has reached 327, expanding the known diversity of both single-nucleotide substitutions and complex loss-of-function alterations.

Sex assignment and the clinical management of individuals with DSD remain subjects of significant debate, particularly concerning 46,XY individuals carrying pathogenic *NR5A1* variants who are raised as females (11). Individuals assigned female at birth with residual testicular function may experience virilization if gonadectomy is not performed, whereas those raised as boys often undergo spontaneous puberty (23). Historically, most of these 46,XY female individuals underwent gonadectomy due to a limited understanding of DSD etiology. Alternatively, some were diagnosed with partial androgen insensitivity syndrome (PAIS) without molecular genetic confirmation and raised as girls (11). The decision to perform a gonadectomy in patients with pathogenic *NR5A1* variants is more complex because the risk of gonadal germ cell tumors varies widely based on multiple factors (36). A higher risk of malignancy is associated with poor testicular differentiation or streak-like dysgenetic gonads (37), ranging from 15% to 50% (38). If a testicular biopsy reveals germ cells and functional seminiferous tubules, early counseling regarding sperm or tissue cryopreservation should be prioritized during or shortly after puberty (39). In female patients with testes, gonadectomy should only be performed after confirming the presence of germ cell neoplasia via biopsy (36). Additionally, an ultrasound screening for congenital splenic anomalies should be considered in individuals with confirmed *NR5A1* variants, given their potential predisposition to severe bacterial infections (40).

Mouriquand et al. (41) emphasized that gender assignment in DSD depends on four critical dimensions: internal (biological/genetic) sex, external anatomy, functional (reproductive) capacity, and social (cultural/educational) factors. However, social sex assignment can conflict with an individual's developing personal and behavioral gender identity. This challenge is illustrated by Song et al. (23), who reported that 16 out of 19 (84.2%) phenotypic girls with an *NR5A1* variant ultimately underwent female-to-male gender reassignment — including five who experienced spontaneous puberty without prior gonadectomy. Similarly, Kumar et al. (42) observed female-to-male gender reassignment in 4 out of 7 (57.1%) patients, with their systematic review confirming this shift in 27 out of 166 (16.3%) cases. To optimize outcomes across domains such as fertility, mental health, and quality of life, Wisniewski et al. (43) echoed the consensus guidelines (2, 3), advocating for a comprehensive, multidisciplinary team approach.

In our case, the patient was assigned a male sex based on clinical counseling and the father's distinct male phenotype. Given the father's successful use of assisted reproduction, this assignment provides the proband with a significant reproductive advantage through future IVF. While long-term psychosexual outcomes into adulthood cannot be predicted, the male sex assignment remains highly appropriate, considering the paternal characteristics.

However, clinical management presented notable timing challenges, as surgical intervention on the patient's genitalia was performed before genetic testing was available. This case underscores how delayed diagnosis increases clinical burdens, requiring the child to undergo complex genital reconstruction to improve his quality of life. Ultimately, these findings highlight the critical necessity of early molecular diagnosis, timely multidisciplinary intervention, and preconception genetic counseling. Fortunately, advances in molecular genetics now allow for rapid diagnosis in such cases, establishing a solid foundation for decisions on the next course of action, including the type and timing of surgery.

## Conclusion

The pathogenic NM_004959.5(NR5A1):c.614dup p.(Gln206Thrfs*20) variant has previously been reported only in 46,XY female patients with DSD. Here, we report a 46,XY male patient who inherited this *NR5A1* variant from his affected father and presented with genital malformations. The father, who exhibited a similar phenotype prior to genital reconstruction, successfully achieved biological fatherhood through *in vitro* fertilization (IVF). Remarkably, the patient did not present with dysgenetic gonads—a feature typically associated with pathogenic *NR5A1* variants—but instead possessed palpable testes. These novel findings demonstrate that individuals harboring a pathogenic *NR5A1* variant can present with genital malformations and testes while retaining their reproductive capacity utilizing assisted reproductive technologies, such as IVF.

## Data Availability

The datasets presented in this study can be found in online repositories. The names of the repository/repositories and accession number(s) can be found below: https://databases.lovd.nl/shared/variants/0000985283#00014792, ID: NR5A1_000120.
