# Supplementary material for: Father and son with a pathogenic variant c.614dup p.(Gln206Thrfs*20) in the NR5A1 gene: a case report
Source: Front Pediatr. 2026 Sep 18;14:1946666. doi: 10.3389/fped.2026.1946666 (PMC13630777; doi:10.3389/fped.2026.1946666)
Supplement: Supplementary file 1 [file Supplementaryfile1.docx]

**Supplementary Material 1:** Virtual panel of 130 genes associated with disorders of sex development.

*AKAP2, AKR1C1, AKR1C2, AKR1C4, AMH, AMHR2, ANOS1, AR, ARL6, ARX, ATF3, ATRX, BBS1, BBS10, BBS12, BBS2, BBS4, BBS5, BBS7, BBS9, BMP15, BMP4, BMP7, BMPR1B, BNC2, CBX2, CCNQ, CDKN1C, CUL4B, CXorf6, CYB5A, CYP11A1, CYP11B1, CYP11B1, CYP17A1, CYP19A1, CYP21A2, DHCR7, DHH, DHX37, DMRT1, DMRT2, DUSP6, ESR2, FEZF1, FGF17, FGF8, FGF9, FGFR1, FGFR2, FLRT3, FOXL2, FSHB, FSHR, GATA4, GHR, GLI2, GNAS, GNRH1, GNRHR, HDAC8, HESX1, HFE, HFM1, HOXA13, HOXA4, HOXB6, HS6ST1, HSD17B2, HSD17B3, HSD17B4, HSD3B2, CHD7, IL17RD, INSL3, KAL1, KISS1, KISS1R, LEP, LEPR, LHB, LHCGR, LHFPL5, LHX1, LHX3, LHX9, MAMLD1, MAP3K1, MCM9, MID1, MKKS, NELF, NLGN4X, NMT2, NR0B1, NR3C1, NR5A1, NSMF, NUP107, PAX2, PCSK1, POLR3A, POR, PROK2, PROKR2, PROP1, PSMC3IP, RSPO1, RXFP2, SEMA3A, SOX10, SOX2, SOX3, SOX9, SPRY4, SRD5A2, SRY, STAR, TAC3, TACR3, TDRD7, TRIM32, TSPYL1, TTC8, TUBB3, WDR11, WNT4, WT1, WWOX, ZFPM2*
